# Overexpression of housekeeping gene *FveIPT2* enhances anthocyanin and terpenoid accumulation in strawberry fruits with minimal impact on plant growth and development

**DOI:** 10.1093/hr/uhaf130

**Published:** 2025-05-26

**Authors:** Lijun Gan, Manman Wei, Shanqi Cao, Hui Zhang, Xuechun Wang, Mingjia Chen, Na Yang, Changhua Zhu, Yi Li

**Affiliations:** College of Life Sciences, Nanjing Agricultural University, No. 6 Tongwei Road, Xuanwu District, Nanjing 210095, China; College of Life Sciences, Nanjing Agricultural University, No. 6 Tongwei Road, Xuanwu District, Nanjing 210095, China; College of Life Sciences, Nanjing Agricultural University, No. 6 Tongwei Road, Xuanwu District, Nanjing 210095, China; College of Life Sciences, Nanjing Agricultural University, No. 6 Tongwei Road, Xuanwu District, Nanjing 210095, China; College of Life Sciences, Nanjing Agricultural University, No. 6 Tongwei Road, Xuanwu District, Nanjing 210095, China; College of Life Sciences, Nanjing Agricultural University, No. 6 Tongwei Road, Xuanwu District, Nanjing 210095, China; College of Life Sciences, Nanjing Agricultural University, No. 6 Tongwei Road, Xuanwu District, Nanjing 210095, China; College of Life Sciences, Nanjing Agricultural University, No. 6 Tongwei Road, Xuanwu District, Nanjing 210095, China; Department of Plant Science and Landscape Architecture, University of Connecticut, Storrs, CT 06269, USA

## Abstract

Anthocyanins and terpenoids are secondary metabolites with well-documented health benefits. Isopentenyl transferases (IPTs) are key enzymes in cytokinin (CK) biosynthesis. While ADP/ATP-type IPTs and their associated *trans*-zeatin (*t*Z)-CKs and iP-CKs are considered to play regulatory roles in growth and development, as well as stress acclimation in plants, tRNA-type IPTs and *cis*-zeatin CKs (*c*Z-CKs), which may serve housekeeping functions, remain less studied. In this study, the tRNA-type IPT gene *FveIPT2* was overexpressed in woodland strawberries (*Fragaria vesca*). Overexpression had minimal impact on plant growth and CK levels but resulted in transgenic fruits exhibiting a significant increase in total phenolic, flavonoid, and anthocyanin contents, indicating enhanced fruit quality. Metabolite profiling revealed substantial increases in nine specific anthocyanins and 24 out of 47 detected terpenoids in the transgenic fruits. Real-time quantitative polymerase chain reaction (RT-qPCR) analysis confirmed the upregulation of genes involved in anthocyanin and terpenoid biosynthesis and transport. These findings demonstrate that while tRNA-type IPTs may primarily play housekeeping roles, *FveIPT2* overexpression can significantly enhance fruit quality by boosting terpenoid and anthocyanin accumulation, highlighting the unexpected potential of these genes to improve the nutritional value of edible fruits.

## Introduction

Cytokinins (CKs) are essential plant hormones that modulate numerous facets of plant growth and developmental processes. These functions include controlling apical dominance, promoting shoot and root development, regulating leaf senescence, and facilitating fruit formation [1–3]. In higher plants, the main types of CKs are zeatin (in *trans* and *cis* isomers, abbreviated as *t*Z and *c*Z) and isopentenyl adenine (iP), along with their riboside conjugates (*t*ZR, *c*ZR, iPR) [2, 4].

Isopentenyl transferases (IPTs), which attach an isopentenyl group to an adenosine molecule, catalyze the first committed step of CK production [2]. IPTs exist in two forms: adenylate-type IPTs (ATP/ADP-type IPTs) and transfer RNA-type IPTs (tRNA-type IPTs), with ATP/ADP and tRNA serving as their respective adenine substrates [4, 5]. A particular adenine at position 37 on the antiloop of tRNAs that identify codons starting with uracil is the target of tRNA-type IPTs [4]. In *Arabidopsis*, there are nine *IPT* genes (*AtIPT1*–*9*), with seven (*AtIPT1, 3, 4*–*8*) encoding ATP/ADP-type IPTs and the remaining two (*AtIPT2* and *AtIPT9*) encoding tRNA-type IPTs [5]. The *atipt1,3,5,7* quadruple mutant significantly decreases the levels of iP-CKs and *t*Z-CKs, demonstrating that ATP/ADP-type IPTs are responsible for generating both types of CKs [5]. Mutations in *atipt2*, *atipt9*, or the *atipt2,9* double mutant exert negligible influence on *t*Z- or iP-CKs concentrations yet greatly impact *c*Z-CK levels [5, 6]. This implies that the bulk of *c*Z-CKs production likely stems from the degradation of prenylated tRNA [5].

Previous studies have established that *t*Z- and iP-CKs are the main biologically active CKs in plants, while *c*Z has shown lower activity [4, 7, 8]. However, ongoing research has uncovered significant levels of *c*Z-CKs in specific crops such as rice [9] and maize [10, 11], as well as during certain developmental stages associated with restricted growth [4, 11, 12]. Intriguingly, maize roots contain a much higher concentration of *c*Z-CKs compared to iP- and *t*Z-CKs combined [4, 10]. The reasons behind this elevated *cZ-CKs* content in these plants remain unknown. While the roles of *t*Z- and iP-CKs in plants have been extensively explored, the functions of *c*Z-type CKs remain less studied [4].

In our previous study [13], we demonstrated that tRNA-type *IPTs* are highly conserved, consistently retained, and constitutively expressed across diverse tissues and environmental conditions in angiosperms. In contrast, ATP/ADP-type *IPTs* have undergone significant expansion and functional divergence, exhibiting tissue- or organ-specific expression patterns and responsiveness to environmental stresses. Based on these findings, we hypothesized that tRNA-type *IPT* genes, along with their associated *c*Z-CKs, serve an essential housekeeping role in plants, while ATP/ADP-type *IPT* genes and their iP/*t*Z-CK counterparts are involved in regulatory functions related to growth and development, as well as stress acclimation in plants [13]. However, the functional characterization of *c*Z-CKs and tRNA-type *IPT* genes is still incomplete, requiring further experimental evidence to confirm this hypothesis.

Housekeeping genes typically support essential cellular functions rather than directly influencing growth, developmental timing, or stress responses. This raises the question: could overexpressing a housekeeping tRNA-type *IPT* gene impact specific metabolic pathways? Anthocyanins, the primary pigments in strawberry fruits [14], and terpenoids—essential secondary metabolites in plants [15, 16]—both contribute to fruit quality and nutritional value. These secondary metabolites are well known for their various health benefits [17–19]. Studies have shown that CK can induce anthocyanin accumulation across various plant species, including *Arabidopsis* [20], apple [21], strawberry [22], and *Eucalyptus* [23], and regulate terpenoid biosynthesis in *Artemisia alba* [24] and *Thymus vulgaris* [25]. Given the health benefits of anthocyanins and terpenoids, we explored whether manipulating a housekeeping *IPT* gene could enhance the production of anthocyanins and terpenoids.

The woodland strawberry (*Fragaria vesca*) has emerged as a valuable model plant for investigating fruit development in other members of the *Rosaceae* family. This is due to its short growth cycle, availability of its genome sequence, and ease of gene editing [14, 26–28]. In *F. vesca*, two genes, *FveIPT2* and *FveIPT7*, belong to the tRNA-type *IPT* family, while five genes, *FveIPT1* and *FveIPT3–6*, are classified as ATP/ADP-type *IPTs* [29]. To gain deeper insights into the role of tRNA-type *IPT* in fruit quality, we overexpressed *FveIPT2* in woodland strawberries. Surprisingly, overexpression of *FveIPT2* has minimal impact on the levels of CKs. However, it leads to a significant elevation of the concentrations of terpenoids and anthocyanins, which are secondary metabolites associated with fruit quality and pigmentation. These findings provide experimental evidence for the roles of tRNA-type *IPT* genes and enhance our understanding of the regulatory mechanisms underlying secondary metabolite synthesis in strawberry fruits.

## Results

### Overexpression of *35S::FveIPT2* led to a slight rise in cytokinin levels

**Figure 1:**
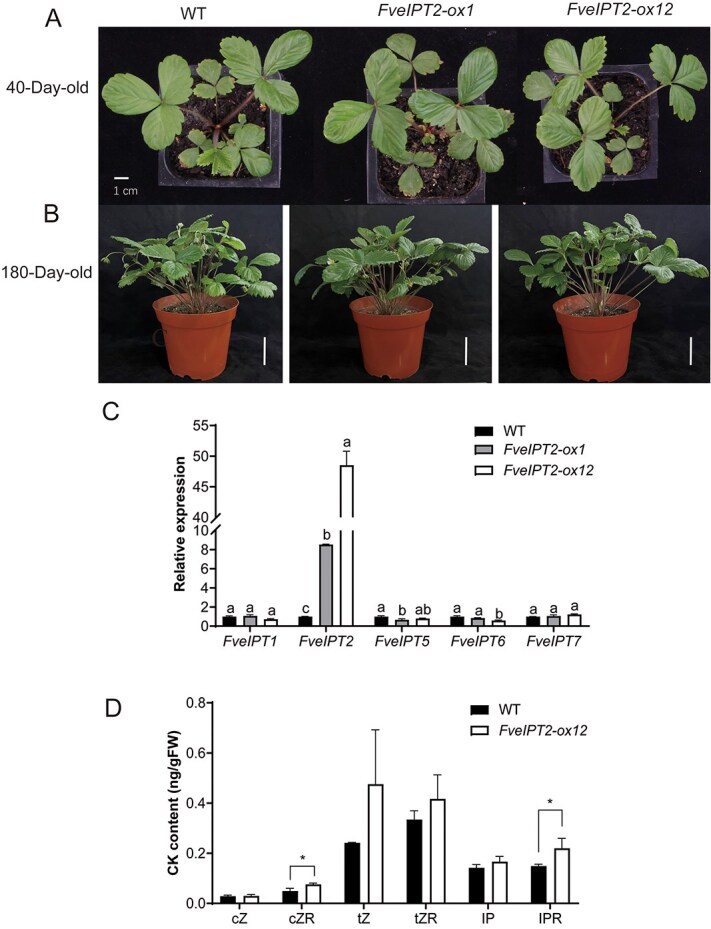
Overexpression of *35S::FveIPT2* in strawberry has minimal impact on plant growth, morphology, and cytokinin levels. No noticeable effects were observed in the *35S::FveIPT2* transgenic plants (40-day-old seedlings were shown in A and 180-day-old seedlings were shown in B). WT: wild type; *FveIPT2-ox1* and *FveIPT2-ox12* are *35S::FveIPT2* transgenic plants. Bar = 5 cm in (B). (C) *FveIPT2* was expressed at high levels in 40-day-old transgenic seedlings. Error bars are SD (*n* = 3). *P* < 0.05. (D) A slight elevation in the concentrations of certain CKs was observed in 40-day-old transgenic seedlings. Error bars are SD (*n* = 3). *P* < 0.05.

To elucidate the function of the tRNA-type *IPT* gene, *FveIPT2*, it was overexpressed using the promoter of cauliflower mosaic virus (CaMV) 35S in *F. vesca*. Two independent transgenic lines, denoted as *FveIPT2-ox1* and *FveIPT2-ox12*, were selected for further analysis. As shown in Fig. 1A and B, no apparent morphological changes were observed in any of the transgenic lines. The relative expression levels of *FveIPT2* in 40-day-old seedlings between the transgenic lines and wild type (WT) were compared utilizing real-time quantitative polymerase chain reaction (RT-qPCR). The findings revealed a marked upregulation of *FveIPT2* expression in *FveIPT2-ox1* and *FveIPT2-ox12*, with expression levels 8.54 and 48.5 times higher than those in the WT, respectively. However, minor alterations were observed in the expression levels of other *FveIPT* genes (Fig. 1C). Owing to the previously reported low expression levels of *FveIPT3* and *FveIPT4*, we were unable to measure their expression in seedlings [29].

In order to assess the impact of *FveIPT2* overexpression on CK levels, we measured CK levels in 40-day-old seedlings of the transgenic line *FveIPT2-ox12* and WT using UHPLC-TQ-MS/MS (ultra-high-performance liquid chromatography coupled with triple quadrupole electrospray tandem mass spectrometry). The findings revealed that the overexpression of *FveIPT2* resulted in a slight elevation in the concentrations of certain CKs such as *c*ZR and iPR (Fig. 1D). These CKs are products of the degradation of prenylated tRNA.

### Overexpression of *35S::FveIPT2* increased fruit quality

**Figure 2:**
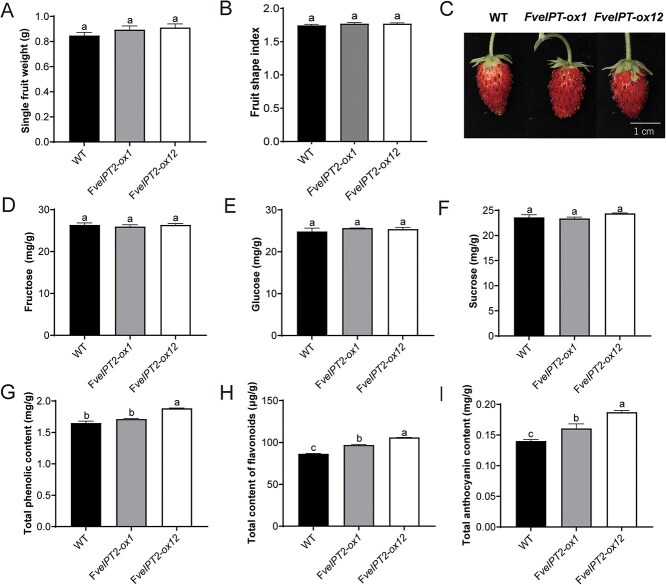
Overexpression of *35S::FveIPT2* in strawberry enhanced fruit quality. Measurements of single fruit weight (A) and fruit shape index (B). (C) Fruit phenotype. Overexpression of *FveIPT2* did not affect the levels of fructose (D), glucose (E) or sucrose (F). Overexpression of *FveIPT2* elevated the levels of total phenolic (G), total flavonoid (H) and total anthocyanin (I). Error bars are SD (*n* = 3). *P* < 0.05.

To investigate the impact of overexpression of *35S::FveIPT2* on fruit growth and development, we compared the vegetative growth phase (from seed germination to first flowering) and the duration from anthesis to fruit ripening between transgenic and WT plants. Both developmental stages showed no statistically significant differences between transgenic and WT plants (Supplementary Fig. S1). Furthermore, measurements of single fruit weight and fruit shape revealed no significant changes in these parameters in transgenic fruits compared with WT (Fig. 2A and B). However, transgenic fruits exhibited a slightly darker red coloration compared with WT fruits (Fig. 2C). Further analysis showed that the overexpression of *35S::FveIPT2* had no significant impact on the concentrations of soluble sugars, such as fructose, glucose, and sucrose. However, it led to a marked elevation in the accumulation of total anthocyanin, total phenolic, and total flavonoid contents, thereby enhancing fruit quality (Fig. 2D–I). For example, relative to WT, the total anthocyanin content in *FveIPT2-ox1* and *FveIPT2-ox12* was 1.15-fold and 1.34-fold, respectively (Fig. 2I).

### Comprehensive metabolite profiling of red receptacles

To comprehensively analyze the changes in metabolites influenced by the overexpression of *FveIPT2*, the receptacles of *FveIPT2-ox12* and WT at the red fruit stage were examined using the quasi-targeted metabolomics technique. The principal component analysis (PCA) plot demonstrated that *FveIPT2-ox12* showed distinct separation in PC1 compared to the WT, indicating altered metabolite accumulation in the transgenic fruits (Supplementary Fig. S2A). In total, 1058 metabolites were identified in these samples (Supplementary Table S1), which were further categorized into 21 types including 47 terpenoids, 194 amino acids and their derivatives, 150 flavonoids, and other categories (Supplementary Fig. S2B).

**Figure 3:**
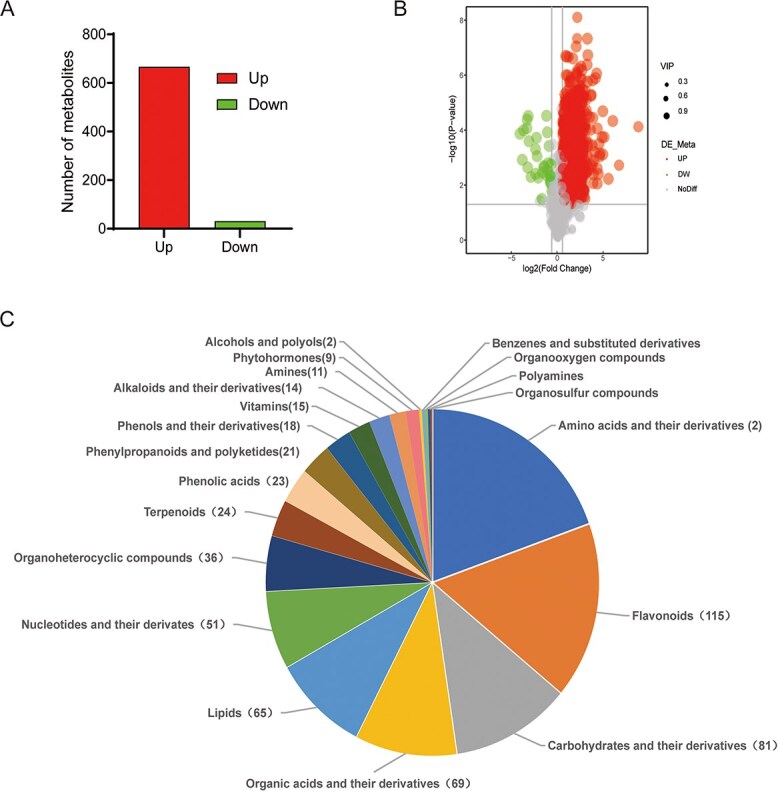
The levels of amino acids and their derivatives, and flavonoids were significantly altered in the *FveIPT2-ox12* transgenic fruits. (A) 666 metabolites were increased and 31 metabolites were reduced in transgenic fruits. Red-stage fruits (without seeds) from both WT and *FveIPT2-ox12* plants were used for quasi-targeted metabolomics analysis. The criteria used to identify DEMs were as follows: VIP > 1.0, FC > 1.5 or FC < 0.667 and *P*-value < 0.05. (B) Volcano plot of DEMs between *FveIPT2-ox12* and WT. The size of each dot reflects the VIP value. (C) The classification of DEMs revealed substantial alterations in amino acids and their derivatives, as well as flavonoids, in the fruits of *FveIPT2-ox12* compared to those of WT. The numbers in parentheses indicate the counts of DEMs.

Using specific criteria (VIP > 1.0, FC > 1.5 or FC < 0.667, and *P*-value <0.05), we identified a total of 697 differentially expressed metabolites (DEMs) when comparing *FveIPT2-ox12* to the WT. Among these DEMs, 666 metabolites showed increased abundance, while 31 metabolites exhibited reduced levels (Supplementary Table S1; Fig. 3A and B). These compounds were classified as amino acids and their derivatives (136), flavonoids (115), carbohydrates and their derivatives (81), and others (Fig. 3C).

### Anthocyanins were significantly increased in the transgenic fruits

**Figure 4:**
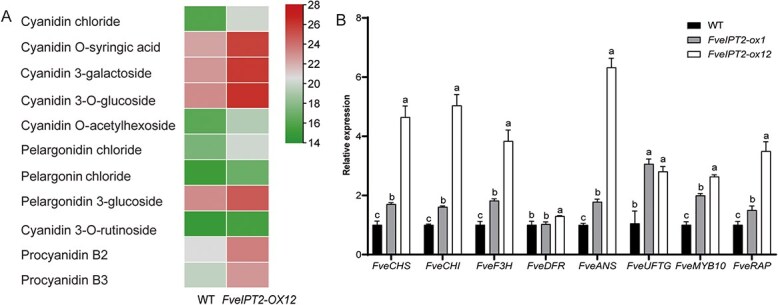
Overexpression of *35S::FveIPT2* in strawberry enhanced anthocyanin production. (A)Anthocyanin production was enhanced in transgenic fruits. Red-stage fruits (without seeds) from both WT and *FveIPT2-ox12* plants were subjected to quasi-targeted metabolomics analysis. Each value in the heatmap represents the log_2_-transformed original value of the anthocyanins. (B) The expression levels of anthocyanin-related genes were elevated in fruits from transgenic plants. Pre-turning stage fruits (without seeds) were used for RT-qPCR analysis, with *FveCHC* serving as the internal reference. Error bars are SD (*n* = 3). *P* < 0.05.

Flavonoids constitute one of the largest categories of secondary metabolites found in fruits. They not only contribute to the flavor and sensory properties of fruits but also serve as powerful antioxidants in the human diet [30]. Anthocyanins, a subclass of water-soluble flavonoids, are particularly important for determining the color, fragrance, and taste profile of fruits [14, 31]. In our metabolomics study, we observed significant changes in 115 flavonoid compounds in *FveIPT2* transgenic fruits (Fig. 3C). Out of these, nine anthocyanins exhibited a substantial increase in abundance in *FveIPT2-ox12* fruits compared to the WT. These included cyanidin chloride (18.2-fold), cyanidin O-syringic acid (9.96-fold), cyanidin 3-galactoside (9.57-fold), cyanidin 3-O-glucoside (9.16-fold), cyanidin O-acetylhexoside (8.22-fold), pelargonidin chloride (6.82-fold), pelargonin chloride (3.25-fold), pelargonidin 3-glucoside (3.27-fold), and cyanidin 3-O-rutinoside (1.52-fold) (Fig. 4A). Additionally, two procyanidins (procyanidin B2 and procyanidin B3) were also found to be elevated in fruits from *FveIPT2-ox12* (Fig. 4A).

To further explore the effect of *FveIPT2* overexpression on anthocyanin accumulation, receptacles at the pre-turning stage were used to analyze the expression of genes related to anthocyanin production, as this is the stage when anthocyanin accumulation initiates [32]. Our RT-qPCR data, in line with the metabolic findings, demonstrated significantly elevated expression levels of several genes associated with the pathways of anthocyanin biosynthesis and transport in the transgenic fruits compared to the WT. Key anthocyanin structural genes such as *FveCHS*, *FveCHI*, *FveF3H*, *FveANS*, and *FveUFTG* were notably upregulated in the transgenic fruits (Fig. 4B). FveRAP performs a crucial role in anthocyanin transport in strawberry fruits [33, 34]. The expression levels of *FveRAP* in *FveIPT2-ox1* and *FveIPT2-ox12* were 1.5 and 3.5 times higher, respectively, than those in the WT (Fig. 4B). Furthermore, the MYB transcription factor FveMYB10, known for its pivotal role in regulating anthocyanin production in fruits [35], showed increased expression in the transgenic fruits (Fig. 4B).

### Overexpression of *35S::FveIPT2* in strawberry enhanced terpenoid accumulation in fruits

**Figure 5:**
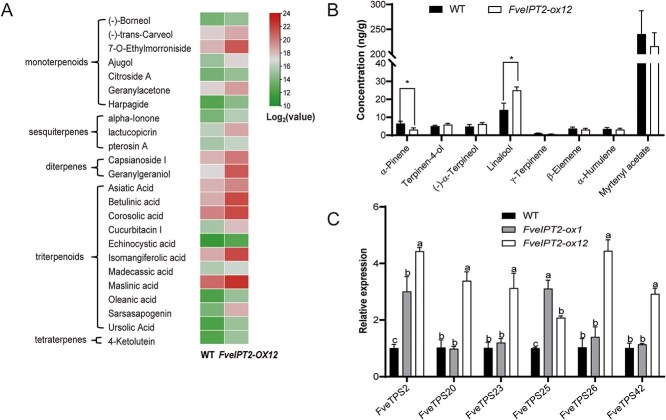
Overexpression of 35S::*FveIPT2* in strawberry elevated terpenoid accumulation in fruits. (A) Terpenoid production was elevated in transgenic fruits. Red-stage fruits (without seeds) from both WT and *FveIPT2-ox12* plants were subjected for quasi-targeted metabolomics analysis. Each value in the heatmap represents the log_2_-transformed original value of terpenoids. (B) Linalool content was elevated, while α-pinene was decreased in *FveIPT2-ox12* fruits. The volatile terpenoid contents in red fruits of both *FveIPT2-ox12* and WT were detected by GC–MS. *P* < 0.05. (C) The expression levels of several genes associated with terpenoid production were significantly altered in transgenic fruits. Pre-turning stage fruits (without seeds) were used for RT-qPCR analysis, with *FveCHC* serving as the internal reference. Error bars are SD (*n* = 3). *P* < 0.05.

Compared with the WT, *FveIPT2-ox12* fruits exhibited significantly higher levels of terpenoids. Among the 47 terpenoids detected in these samples, 24 terpenoids showed a marked elevation in the fruits of *FveIPT2-ox12*, including 11 triterpenoids, 2 diterpenoids, 3 sesquiterpenoids, 7 monoterpenoids, and 1 tetraterpene (Fig. 5A). The top five terpenoids with the highest increase in *FveIPT2-ox12* were identified as sarsasapogenin, geranylgeraniol, betulinic acid, isomangiferolic acid, and 7-o-ethylmorroniside. Their levels in *FveIPT2-ox12* were 25.55, 16.86, 10.12, 9.83, and 8.19 times higher than those in the WT, respectively (Fig. 5A).

Volatile terpenoids represent the largest family of plant volatile compounds. Therefore, we analyzed the concentrations of volatile terpenes in the receptacles of both *FveIPT2-ox12* and WT. The results showed that *FveIPT2-ox12* had a significantly lower amount of α-pinene compared to the WT. In contrast, *FveIPT2-ox12* exhibited a higher level of linalool than the WT (Fig. 5B). Terpinene-4-ol, (−)-α-terpineol, γ-terpinene, β-elemene, α-humulene, and myrtenyl acetate were also detected in strawberry receptacles, but their concentrations did not show significant alterations in the fruits of *FveIPT2-ox12* (Fig. 5B).

Terpene synthase (TPS) enzymes catalyze the key step in the conversion of terpenoid precursors into various terpenoid compounds [36, 37]. We analyzed the expression of several genes encoding TPS that have been previously reported to be highly expressed in strawberry receptacles [37]. We observed that these *TPS* genes were significantly upregulated in the fruits of *FveIPT2-ox12* compared with the WT (Fig. 5C).

### Type-A response regulator genes were significantly downregulated in transgenic fruits

**Figure 6:**
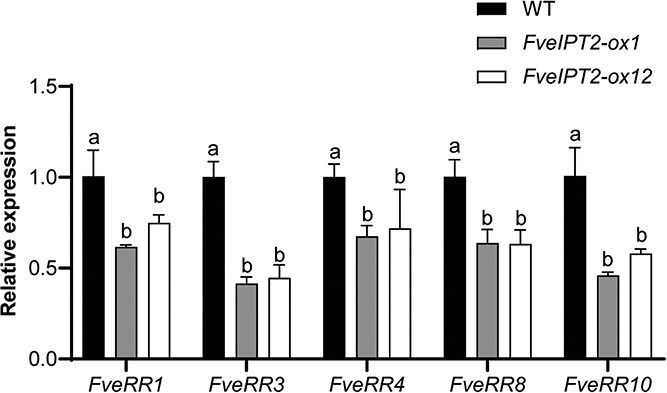
Altered expression levels of CK-regulated genes in *35S::FveIPT2* transgenic fruits. CK-responsive genes were downregulated in transgenic fruits. Pre-turning stage fruits (without seeds) were used for RT-qPCR analysis, with *FveCHC* serving as the internal reference. Error bars are SD (*n* = 3). *P* < 0.05.

Type-A response regulator (ARR) genes, which are rapidly induced by CK and serve as marker genes for CK signaling pathway [2, 7], were measured to investigate whether the increased levels of anthocyanins and terpenoids in fruits overexpressing *FveIPT2* are related to the activation of CK signaling. We measured the expression levels of three *ARR* genes, namely *FveRR1*, *FveRR3*, and *FveRR4*, in the receptacles. Interestingly, these marker genes were significantly downregulated in transgenic fruits. Additionally, two type-B RR genes, namely *FveRR8* and *FveRR10*, were also downregulated in the transgenic fruits (Fig. 6). These findings indicate that the increased contents of anthocyanins and terpenoids in *FveIPT2* overexpressing fruits may not be directly associated with type-A RR-mediated CK signaling pathway.

## Discussion

Housekeeping genes are characterized as those that exhibit consistent expression across various tissues, are essential for fundamental cellular maintenance functions, and are evolutionarily conserved across diverse species [38]. Previously, we proposed that tRNA-type *IPT* genes serve a fundamental housekeeping role in plants, while ATP/ADP-type *IPTs* are involved in regulatory functions related to growth and development, as well as stress acclimation in plants [13]. Further experimental validation is required to substantiate this hypothesis. While the phenotypic effects of manipulating ATP/ADP-type *IPTs* have been widely studied, the effects of altering tRNA-type *IPTs*, particularly through overexpression, remain largely unexplored [4].

In this study, we overexpressed *FveIPT2* in woodland strawberries using the 35S CaMV promoter. Although *FveIPT2* expression was significantly elevated in transgenic plants compared with the WT, this overexpression led to merely a slight elevation in the overall concentrations of *c*ZR and iPR in young seedlings (Fig. 1). However, because our current method measures total CK levels across all analyzed tissue types, we cannot rule out the potential for significant changes in CK levels within specific tissues. Recently, the knockout of two tRNA-type *IPT* genes, *atipt2* and *atipt9*, in *Arabidopsis* revealed distinct effects on *c*Z-CKs levels in siliques: the *atipt2* mutant exhibited reduced total *c*Z-CKs, while the *atipt9* mutant showed increased levels compared with the WT [6]. These findings suggest that *c*Z-CKs synthesis in plants is a complex, finely regulated process, likely influenced by tissue- and organ-specific factors that affect post-transcriptional regulation.

In tomato, constitutive expression of *SIIPT3*, an adenylate-type *IPT* gene, resulted in the overproduction of CKs (levels increased up to 12-fold) and significant alterations in plant phenotype such as dwarfism, and branchy and ligneous stems [39]. Similar effects were reported in *Arabidopsis* when an adenylate-type *IPT* gene, *ZmIPT2*, was overexpressed [40]. However, in the present study, the overexpression of *FveIPT2* in strawberry had minimal impact on plant growth, morphology, and CK levels (Fig. 1). The lack of phenotypic changes associated with *FveIPT2* overexpression stands in stark contrast to the significant morphological alterations typically observed when adenylate-type IPT genes are overexpressed [39, 40]. Housekeeping genes are defined by their essential roles in maintaining basic cellular functions. They are consistently expressed across all cell types under both normal and stress conditions, rather than being involved in the regulation of developmental processes or environmental responses [38]. The unchanged morphology, developmental progression, and growth patterns in *FveIPT2*-overexpressing plants further support the hypothesis that tRNA-type *IPT* genes function as housekeeping genes in higher plants [13].

Despite minimal effects on CK levels and plant growth, *FveIPT2* overexpression significantly elevated anthocyanin content in transgenic fruits. Nine anthocyanins, including six cyanidin derivatives and three pelargonidin derivatives, accumulated at higher levels in transgenic fruits accompanied by upregulated expression of biosynthetic (*FveCHS*, *FveCHI*, *FveF3H*, *FveANS*, and *FveUFTG*), regulatory (*FveMYB10*), and transport-related (*FveRAP*) genes (Fig. 4). Similarly, various terpenoids, including monoterpenoids, sesquiterpenoids, diterpenoids, triterpenoids, and tetraterpenoids, were elevated in the transgenic fruits, with altered expression of *TPS* genes in *FveIPT2* overexpression plants (Fig. 5A). Linalool, which is one of the most important terpenoids in strawberries, possesses a sweet, floral, and citrus-like aroma that enhances the fruit’s fragrance [41]. On the other hand, the olefinic monoterpene α-pinene contributes to the turpentine-like, woody, resinous, and unpleasant odor in strawberries [41]. Interestingly, in the present study, a notable reduction in α-pinene concentration and an elevation in linalool content in the fruits of *FveIPT2-ox12* were observed (Fig. 5B), indicating that the overexpression of the *FveIPT2* gene improves the scent of strawberry fruits.

The inductive effect of *FveIPT2* overexpression on anthocyanin and terpenoid accumulation parallels studies in *Arabidopsis* and *Thymus vulgaris* L, where CKs modulate these metabolites [20, 24, 42, 43]. We explored the underlying mechanisms responsible for the observed metabolic changes. Our analysis of CK-responsive gene expression revealed a marked downregulation of canonical type-A RR-mediated signaling in transgenic plants (Fig. 6). This suggests that traditional CK signaling pathways are unlikely to be the primary drivers of the increased anthocyanin and terpenoid accumulation.

tRNA-IPT catalyzes the prenylation of adenosine-37 within tRNAs, which leads to *c*Z-CK production through the degradation of prenylated tRNAs [6, 44]. In *Arabidopsis*, mutations of tRNA-type *IPT* genes (*AtIPT2* and *AtIPT9)* resulted in developmental defects such as smaller meristem size and reduced primary root elongation, yet exogenous *c*Z fails to rescue these phenotypes, implying direct roles for tRNA modification beyond *c*Z biosynthesis [45]. It is also possible that externally applied *c*Z does not effectively reach the tissues or cells essential for these phenotypes. Further research is needed to explore whether the enhanced fruit quality observed in *FveIPT2*-overexpressing transgenic plants is linked to tRNA modifications.

In conclusion, our study reinforces the housekeeping role of the tRNA-type *IPT* gene *FveIPT2* and demonstrate that its overexpression enhances strawberry fruit quality without compromising growth. The metabolic improvements, mediated through upregulated anthocyanin and terpenoid pathways, highlight *FveIPT2* as a promising target for the genetic improvement of strawberry fruit quality by increasing health-promoting secondary metabolites. Future studies are needed to elucidate the underlying molecular mechanisms.

## Materials and methods

### Plant materials and growth conditions

The woodland strawberry (*F. vesca*) accession ‘Ruegen’ was cultivated in a controlled greenhouse at Nanjing Agricultural University, China, under fluorescent lighting at an intensity of 120 μmol·m^−2^·s^−1^. The environment was controlled at a temperature of 22 ± 2°C, with a 16-h photoperiod and 60% relative humidity. For fruit phenotype analysis, flowers from plants of the same age that bloomed on the same day were selected for labeling and manual pollination to ensure consistency. There were no significant differences between transgenic and WT plants in the time from flowering to fruit ripening (Supplementary Fig. S1); therefore, fruits were harvested 30 days postpollination for analysis.

### Stable transformation of strawberry

To construct the *FveIPT2* overexpression vector, the complete coding sequence (CDS) of *FveIPT2* (FvH4_3g29650) was cloned into the 2300GNR vector using specific primers (Supplementary Table S2), driven by the CaMV35S promoter. The construct was transformed into‘Ruegen’ via *Agrobacterium* (GV301)-mediated transformation performed following the previously described method [33]. Leaf explants were used for transformation, with putative transgenic calli selected through antibiotic resistance screening. Genotypic analysis was performed via PCR using a forward primer specific to the 35S promoter and a reverse primer targeting the coding sequence (Table S2), followed by Sanger sequencing confirmation. T3 plants were used for further analysis because this also demonstrates that the observed phenotypes are heritable and stable after multiple generations of sexual reproduction.

### Extraction, purification, and measurement of CKs

For the analysis of CKs, 40-day-old seedlings from both WT and transgenic plants were harvested. CKs were extracted with 2-propanol/water/concentrated HCl (200:100:0.2, v/v/v). Following 12 h of shaking at 100 rpm (4°C), dichloromethane was added, and the mixture was shaken again. After phase separation, the lower organic phase was filtered through a 0.45-μm membrane. The samples were then dried, redissolved in 200 μl methanol and 200 μl water, and centrifuged (12 000 rpm, 10 min, 4°C). The supernatant was filtered through a 0.22-μm membrane and analyzed using an Agilent 1290 UPLC system (Agilent, USA) coupled with an AB SCIEX QTRAP® 6500 mass spectrometer (Applied Biosystems, USA), following the procedures outlined in our previous study [1].

### Assessment of single fruit weight and fruit size

The weight of fruits was determined using a precision balance. The length and diameter of fruits were assessed using Vernier calipers and the fruit shape index was derived by dividing the fruit length by its diameter.

### Measurement of total anthocyanin, flavonoid, phenolic, and soluble sugars

The total anthocyanin content was quantified using the pH differential method, in accordance with the protocol established by Tonutare *et al*. [46]. The total flavonoid was analyzed using colorimetry described by Xie *et al*. [47] and rutin was used to generate a standard curve. The total phenolic content was assessed according to the methodology described by Yan *et al*. [48]. The results were calculated by a standard curve of gallic acid. The quantification of soluble sugars was performed using HPLC as described by Gu *et al*. [32]. For each analysis, at least 10 fruits at the red stage, excluding seeds, were combined to create a single biological replicate. Three biological replicates were utilized for the analysis.

### Volatile compounds analysis

To measure volatile compounds, samples were prepared using headspace solid-phase microextraction (HS-SPME), and the volatile profiles were characterized by gas chromatography–mass spectrometry (GC–MS) as described by Negri *et al*. [49]. 3-octanol was added to each sample as an internal standard composition. Volatile terpenoids were identified by cross-referencing mass spectra and retention times with both the NIST library (National Institute of Standards and Technology, 2014) and the Human Metabolome Database (HMDB). For each analysis, at least 10 fruits, excluding seeds, were combined to create an individual biological replicate. Three biological replicates were utilized for the analysis.

### RNA extraction and RT-qPCR

Total RNA was isolated from the samples using the cetyltrimethylammonium bromide (CTAB) method, in accordance with the protocol established by White *et al*. [50]. Subsequently, RT-qPCR was conducted following the manufacturer’s guidelines using the extracted RNA. Primers used for RT-qPCR are detailed in Supplementary Table S2.

### Quasi-targeted metabolomics analyses

To analyze the metabolomics profile, fruits (without seeds) at the red stage from both WT and OE12 plants were sent to Novogene Bioinformatics Technology Co., Ltd. (Beijing, China). The analysis of quasi-targeted metabolomics was performed following the methodology reported by Jiang *et al*. [51]. The LC–MS/MS analyses were conducted utilizing an ExionLC™ AD system (SCIEX) in conjunction with a QTRAP® 6500+ mass spectrometer (SCIEX). Metabolite profiling was performed using Multiple Reaction Monitoring (MRM) based on the Novogene in-house database. Metabolite identification was performed using Q1 and Q3 ions, along with retention time, declustering potential, and collision energy. Quantification was conducted using Q3. Raw data were processed with SCIEX OS software (v1.4) for peak integration and correction. Metabolites were annotated using the Kyoto Encyclopedia of Genes and Genomes (KEGG), Human Metabolome Database (HMDB), and LIPID MAPS (https://www.kegg.jp/, https://hmdb.ca/, https://www.lipidmaps.org/). The criteria used to identify DEMs were as follows: VIP (Variable Importance in Projection) score >1.0, fold change >1.5 or <0.667, and a *P*-value < 0.05. Three biological replicates were utilized for the analysis.

### Statistical analyses

All analyses in this report were performed on three independent experiments. Statistical analyses were carried out with GraphPad Prism 8.0.1. (GraphPad, San Diego, CA, USA). Differences between treatments were tested using Duncan’s multiple range test for analysis of variance (ANOVA) with a significant level of *P* < 0.05. Different letters were used to indicate significant differences. Alternatively, a Student’s *t*-test was employed for specific comparisons.

## Supplementary Material

Web_Material_uhaf130

## Data Availability

All relevant data can be found within the manuscript and its supporting material.
