# Supplementary material for: Overexpression of housekeeping gene FveIPT2 enhances anthocyanin and terpenoid accumulation in strawberry fruits with minimal impact on plant growth and development
Source: Hortic Res. 2025 May 26;12(8):uhaf130. doi: 10.1093/hr/uhaf130 (PMC12268167; doi:10.1093/hr/uhaf130)
Supplement: Web_Material_uhaf130 [file web_material_uhaf130.zip › supplementary Fig.S1.docx]

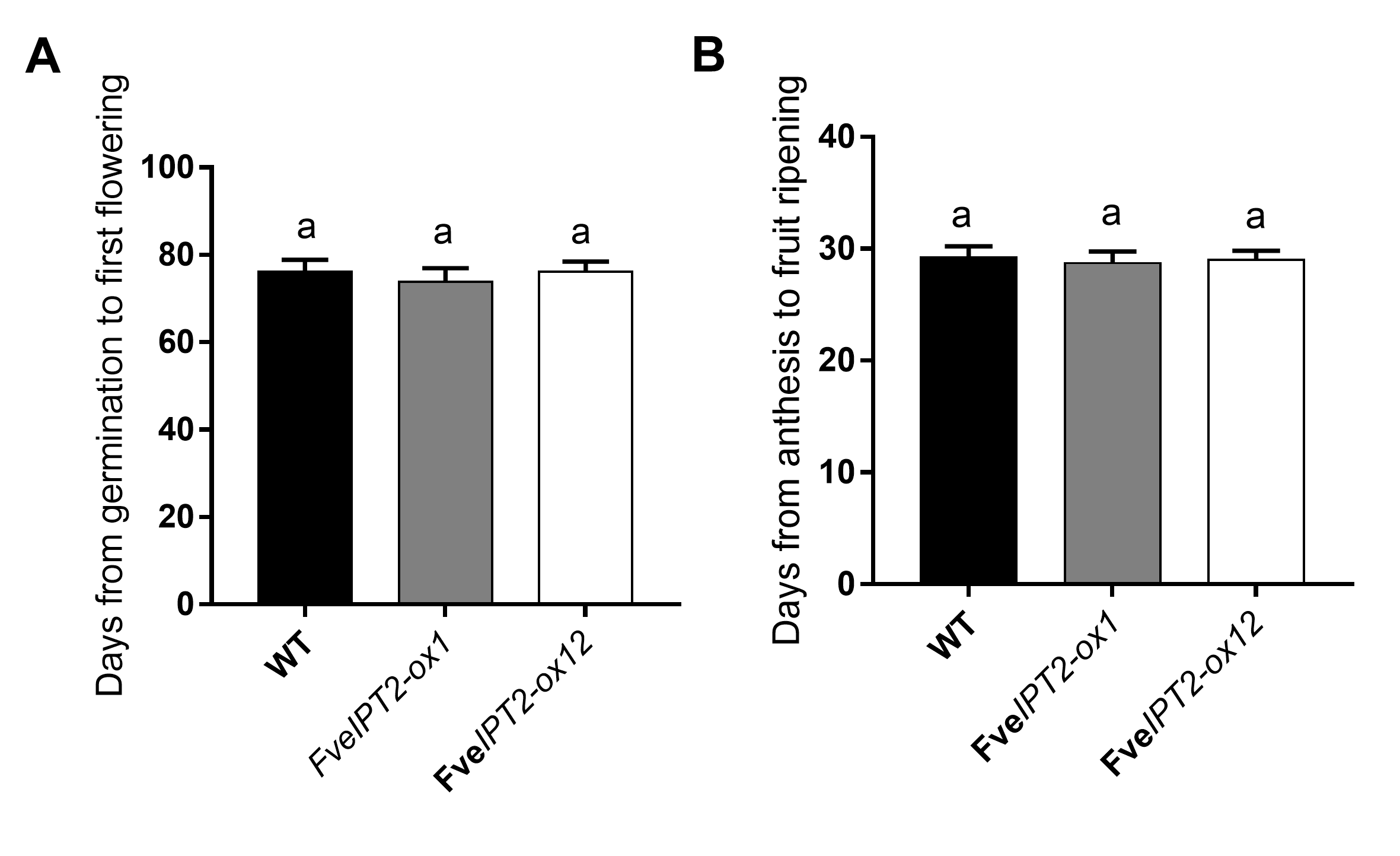


Fig. S1. Effect of overexpression of *35S::FveIPT2* on vegetative growth phase and duration from anthesis to fruit ripening in transgenic and WT plants.

(A) vegetative growth phase (from seed germination to first flowering);

(B) Days from anthesis to fruit ripening.
