# Supplementary material for: Overexpression of housekeeping gene FveIPT2 enhances anthocyanin and terpenoid accumulation in strawberry fruits with minimal impact on plant growth and development
Source: Hortic Res. 2025 May 26;12(8):uhaf130. doi: 10.1093/hr/uhaf130 (PMC12268167; doi:10.1093/hr/uhaf130)
Supplement: Web_Material_uhaf130 [file web_material_uhaf130.zip › supplementary Fig.S2.docx]

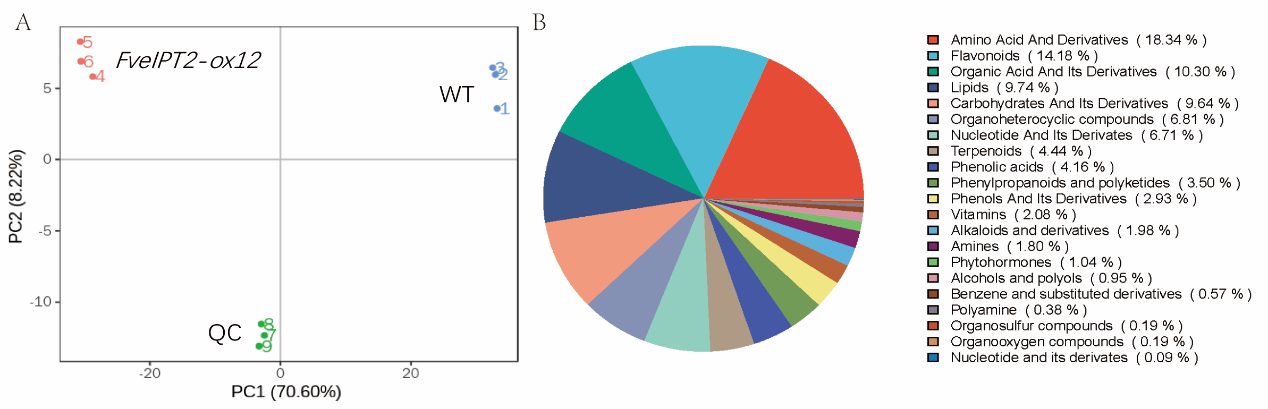


Fig. S2. Effect of overexpression of *35S::FveIPT2* on metabolites in transgenic line *FveIPT2-ox12* and WT.

(A) Principal component analysis (PCA) of metabolites in receptacles of *FveIPT2-ox12* and WT.

(B) Classification of metabolites identified in red receptacles of *FveIPT2-ox12* and WT.
