# Supplementary material for: Overexpression of housekeeping gene FveIPT2 enhances anthocyanin and terpenoid accumulation in strawberry fruits with minimal impact on plant growth and development
Source: Hortic Res. 2025 May 26;12(8):uhaf130. doi: 10.1093/hr/uhaf130 (PMC12268167; doi:10.1093/hr/uhaf130)
Supplement: Web_Material_uhaf130 [file web_material_uhaf130.zip › Supplementary Tables S2.docx]

**Table S2. Primers used in this study**

| **Gene Name** | **Gene ID** | **Forward primer (sequence 5’-3’)** | **Reverse primer (sequence 5’-3’)** | Purpose |
| --- | --- | --- | --- | --- |
| **RT-qPCR** |  |  |  |  |
| *FveCHC* | FvH4_6g53310 | CATCTGTTTCCGCCACAACC  TATACAT | TTTGTTTTTCTCTGAGTTGGC  CATTAGA | RT-qPCR |
| *FveIPT1* | FvH4_4g27230 | GTGGTTCTGGCCGAGTATTTAT | CACTCCGATAGCCTTTCTCAAC |  |
| *FveIPT2*  *FveIPT5*  *FveIPT6*  *FveIPT7*  *FveCHS*  *FveCHI1*  *FveF3H*  *FveDFR*  *FveANS*  *FveUFGT*  *FveMYB10* | FvH4_3g29650  FvH4_2g23840  FvH4_4g14440  FvH4_3g18660  FvH4_7g01160  FvH4_7g25890  FvH4_1g11810  FvH4_2g39520  FvH4_5g01170  FvH4_7g33840  FvH4_1g22040 | CCTGTGGTGACAAGATCCTTAG  CTCTGGGTGGATGTATCCTTATC  GACTCGAACTTCACTGCTACTG  CTGGTATCGTCAGGTCTTTGAC  TGAACATTACCGACTGGAACTC  TCACAACCTCCAAGCCATTAG  TGTCCATAGCGACATTCCAGAACC  CACGATTCACGACATTGCGAAATT  CATTTGGCCTCAAACACCTT  GTGGTCACTTCGGGACAACT  ATGGAGGTGAGAAAAGGTTC | GTGCTGCTCGACAGACAAATA  CAAAGAACTCTCTCACCTCCTC  TCCACCAGTGCCTCTATGTA  AGAGTTCACAGGGTTGCTAATC  ATATGCCTCGTGGCTTCTAAC  CCACAATCTCAGGGTCCAAATA  CTCTGAATCCTGCAGTTGCTCCTT  GAACTCAAACCCCATCTCTTTCAGCTT  CTTCTCCAGCCTCCCTTCTT  AATCAAAGGCACTCCACCTG  GTTTCCCAAAAGCTTCCGAAG |  |
| *FveRAP* | FvH4_1g27460 | GGCTGATCTGAGCCATCTTC | CATGCAGGCCTATTGGAAAT |  |
| *FveTPS2*  *FveTPS20*  *FveTPS23*  *FveTPS25*  *FveTPS26*  *FveTPS42*  *FveRR1*  *FveRR3*  *FveRR4*  *FveRR8*  *FveRR10* | FvH4_1g05400  FvH4_4g27790  FvH4_4g27952  FvH4_5g06470  FvH4_5g06530  FvH4_7g33640  FvH4_4g35230  FvH4_2g27180  FvH4_7g02970  FvH4_4g33470  FvH4_4g19230 | CTGATGCTGCTGCTGATTCT  GCCTTTGTTGGAATGCGAGG  CTGATCTTCACCAGGACCCA  GCCGTTTAAGCCCCTCACTT  TGGCACTTATTACATCCGGCA  AACTGGAGCAACACGTTCAA  AGCCGGTGAAGTTATCGGAC  CCATCTCCTCAGCAGCAGTC  TGTCAACAAGCTTAGACCCCA  TTTGCAGGGGACGAGTTTGA  GATGCTCCGTACTTGTCTCTATG | CGTTCCAGGGCTTGTTCTATT  GGACAGAACTCACTTTGCCC  ATGCCTTGCTTCAACCCTTG  AGCACTGTTGAGTTCCTGGT  CCCAAGTGTGTTTGAAGCCC  GCCATGCCTAAACGTTGAAGG  GAAGGAGGCTCATCGGAAGG  AGATCCCTTGACCCTTTTCAGC  TCATGAAAGCACATCAAAGCCA  TATATTGGGGCGCCATTTGC  GGCATGTGGACGTCACTAATA |  |
|  |  |  |  |  |
| **Gene clone** |  |  |  |  |
| *FveIPT2* | FvH4_3g29650 | GCAGGTCGACTCTAGATGGAG  GGCACTACTGAAAAGC | TTGAACGATCTCTAGCTAACT  ACTGTGCTGCTCGACCT | vector construction |
| **Genotypic analysis** |  |  |  |  |
| *35S::IPT2* |  | GCCCAGCTATCTGTCACTTTAT | CCTTCGGCTTCTCTTCTTCTT |  |
